# 

**Published:** 1907-06-01

**Authors:** 


### BOOKS RECEIVED.

W. H. and L. Collingridge.

"The City of London Directory," 1907.

Baillière, Tindall, and Cox.

"Pulmonary Consumption." Third edition. By A. Latham, M.D.

J. M. Dent and Co.

"Sick Nursing." By H. Drinkwater, M.D. (Temple Primers.)

W. Heinemann.

"Metabolism and Practical Medicine." 2 vols. By Carl Von Noorden.

THE HOSPITAL *June* 1, 1907.

*Name* ..............................................................

*Address* ..............................................................

..............................................................

This Coupon must accompany manuscript or contributions intended for The Hospital.

